# Socioeconomic and regional determinants of optimal antenatal care utilization among women in South and Central Somalia

**DOI:** 10.1016/j.gloepi.2025.100239

**Published:** 2025-12-18

**Authors:** Mohamed Abdirahim Omar, Yahye Sheikh Abdulle Hassan, Abdirasak Sharif Ali, Mohamed Mustaf Ahmed

**Affiliations:** a Faculty of Health Science, Salaam University, Mogadishu, Somalia; b Faculty of Medicine and Health Sciences, Jamhuriya University of Science and Technology, Mogadishu, Somalia; c Department of Microbiology and Laboratory Sciences, Faculty of Medicine and Health Sciences, SIMAD University, Mogadishu, Somalia; d Blood Bank Unit, Banadir Hospital, Mogadishu, Somalia; e SIMAD Institute for Global Health, SIMAD University, Mogadishu, Somalia

**Keywords:** Antenatal care, Somalia, Maternal health, Determinants, Socioeconomic status, Demographic health survey

## Abstract

**Background:**

Somalia faces one of the world's highest maternal mortality ratios, and fewer than one in sixteen pregnant women obtain the previously recommended minimum of four antenatal care (ANC) visits. Understanding the drivers of low ANC use is essential for responsive policy. We investigated the prevalence and determinants of optimal ANC use (≥4 visits) among women in South and Central Somalia using nationally representative survey data.

**Methods:**

We conducted a cross-sectional analysis of the 2020 Somali Health and Demographic Survey. The weighted analytic sample comprised 4124 women aged 15–49 years with complete data. Survey-adjusted descriptive statistics characterized ANC use. Bivariate associations and multivariable survey logistic regression identified independent predictors; adjusted odds ratios (aORs) with 95 % confidence intervals (CIs) are reported.

**Results:**

Only 5.7 % of women (95 % CI 4.3–7.6) had four or more ANC visits. After adjustment, secondary education (aOR 2.33, 95 % CI 1.01–5.40) and higher education (aOR 5.36, 95 % CI 1.58–18.15) were associated with optimal ANC. Household wealth showed a graded increase, with the richest quintile having nearly 30 times the odds compared with the poorest (aOR 29.66, 95 % CI 8.51–101.27). Home delivery was associated with lower odds of optimal ANC (aOR 0.29, 95 % CI 0.18–0.47). Regional disparities persisted: women in Bay (aOR 4.07, 95 % CI 1.22–13.60) and Galgaduud (aOR 2.74, 95 % CI 1.13–6.64) had higher odds than those in Mudug.

**Conclusion:**

Optimal ANC coverage in South and Central Somalia remains critically low. Priorities include reducing financial and geographic barriers to care, strengthening facility-based services, and promoting female education to improve maternal and neonatal outcomes.

## Introduction

Maternal mortality continues to be a major public health concern, particularly in low- and middle-income countries (LMICs), with sub-Saharan Africa being especially affected [1]. Antenatal care (ANC) is essential for enhancing the health of mothers and newborns by facilitating the early identification of potential issues, offering health education, and preparing for childbirth and postnatal care [2,3]. Somalia is grappling with significant maternal health issues, including one of the highest maternal mortality rates globally. According to the 2020 Somali Health and Demographic Survey (SDHS), the maternal mortality ratio is 692 per 100,000 live births, showing a decrease from the 2006 figure of 1044 per 100,000 births [4,5]. Years of chaos, unrest, and a broken healthcare system have made it difficult to obtain good prenatal care [6,7]. The WHO has revised its guidelines to recommend at least eight antenatal care visits, replacing the earlier model of four visits, to enhance the management of pregnancy-related risks and improve maternal outcomes [2,8]. Adherence to the recommended number of antenatal care (ANC) visits is correlated with increased health literacy, the availability of skilled birth attendants, and improved health outcomes for both mothers and newborns [9].

In Somalia, while 85 % of women attend at least one antenatal care visit, only 24 % follow the previous minimum of four visits, with the new WHO recommendation now advising at least eight visits for optimal care [5,10]. South and Central Somalia are particularly affected by years of conflict, political instability, insecurity, and displacement, which have led to limited access to healthcare services [11,12]. These regions face some of the highest maternal mortality rates in the country, exacerbated by underdeveloped health infrastructure and a shortage of skilled health professionals. The specific barriers to accessing adequate healthcare in these regions emphasize the need for targeted interventions to improve ANC. Understanding the regional disparities is crucial for designing effective policies. This study focuses on South and Central Somalia, utilizing the SDHS 2020 data to identify the factors influencing optimal ANC visits. Gaining insights into these determinants will guide the development of policies aimed at enhancing ANC services and improving maternal health outcomes in the country.

## Materials and methods

This study aimed to identify the independent determinants influencing optimal antenatal care (ANC) utilization among women of reproductive age (15–49 years) in South and Central Somalia.

## Data source

This study used the 2020 Somalia Demographic and Health Survey (SDHS) dataset. The SDHS 2020 is a nationally representative survey that gathered extensive health information from women in Somalia and is publicly available. For this analysis, we restricted to women with a live birth in the five years preceding the survey who had complete data on study variables (analytic *n* = 4124). We implemented a complete-case approach; potential implications for selection bias due to non-random missingness are acknowledged in the Limitations.

## Sample size and sampling technique

The SDHS 2020 employed a complex multistage cluster sampling design to ensure national representation. While the original sampling technique involved the selection of clusters and then households within those clusters to reach the study participants, all estimates and models accounted for the DHS survey design using sampling weights, primary sampling units, and stratification to obtain population-representative inferences for women aged 15–49 years in South and Central Somalia. The final analytical sample, as mentioned earlier, comprised complete cases (*n* = 4124).

## Measurement of study variables

### Primary outcome

The primary outcome was optimal ANC utilization, defined as attending ≥4 ANC visits for the most recent live birth in the five years before the survey. Although the 2016 WHO ANC model recommends eight or more contacts, we operationalized ≥4 visits to align with how ANC is measured in the SDHS 2020 and to maintain comparability with prior DHS-based analyses [13,14]. Women who reported fewer than four ANC visits were categorized as having suboptimal ANC utilization.

### Predictors

Drawing on the existing literature and variables available in the SDHS 2020, we examined the following socio-demographic, economic, obstetric, and contextual covariates: maternal age group, educational attainment, marital status, sex and age of the household head, household size, parity/birth order, current work status, household wealth quintile, place of delivery (home vs. facility), region (South/Central regions including Galgaduud, Bay, and Mudug), residence (urban, rural, nomadic), and media exposure.

### Operational definitions

Wealth quintiles followed the DHS asset-based wealth index with survey-weighted cut-off points provided by the DHS (we used DHS-provided quintile categories). Media exposure was coded as “exposed” if the respondent reported radio, television, or newspaper at least weekly; otherwise “not exposed,” matching the terminology used in tables. Household head status and age were obtained from the household roster; household-head age was categorized for analysis (e.g., <30, 30–39, ≥40) to match the contrasts shown in the Results section. Because DHS ANC items pertain to women with a recent live birth, never-married women are rare/absent in the analytic sample; this context is clarified in the Results and table footnotes. Facility-level characteristics and individual geospatial proximity to facilities were not available in the SDHS 2020 and could not be linked at the respondent level.

## Data processing and statistical analysis

The SDHS 2020 data were analyzed using STATA 16. We began with descriptive statistics to characterize the study sample and the prevalence of optimal ANC attendance, reporting survey-weighted counts, percentages, and 95 % confidence intervals for sociodemographic, economic, obstetric, and contextual characteristics. We provide detailed cross-tabulations of outcome counts by covariate levels and report both unadjusted (crude) and adjusted effect estimates (odds ratios with 95 % CIs).

To identify the independent determinants of optimal ANC attendance while controlling for potential confounding factors, we employed a multivariable survey logistic regression model. All variables that were theoretically relevant or showed a statistically significant association (*p* < 0.05) in the bivariate analysis were considered for inclusion in the initial multivariable model. We then used a backward elimination approach to arrive at the final model, retaining variables that had a statistically significant independent effect on optimal ANC attendance at a *p*-value of less than 0.05. Potential multicollinearity among the independent variables was assessed using the Variance Inflation Factor (VIF). As all VIF values were well below the common threshold of 5 (Mean VIF = 1.46, Maximum VIF = 2.85), multicollinearity was not a significant concern for the model. The overall fit of the final logistic regression model was assessed using the F-statistic.

## Results

This section details the findings derived from the analysis of the 2020 Somalia Demographic and Health Survey (SDHS) dataset. The primary objective was to identify the determinants of optimal antenatal care (ANC) utilization, defined as attending four or more ANC visits during the last pregnancy, among women aged 15–49. The final analytical sample comprised 4124 women with complete data for the variables included in the multivariate model.

## Descriptive statistics: sample characteristics and ANC utilization

The overall prevalence of optimal ANC attendance within the analytical sample (*N* = 4124), accounting for the survey design, was estimated to be 5.7 % (95 % CI: 4.3 %–7.6 %). Consequently, 94.3 % (95 % CI: 92.4 %-95.7 %) of pregnant women in this sample reported suboptimal ANC utilization (less than four visits).

## Socio-demographic and obstetric characteristics of pregnant women in the analytical sample

The sociodemographic profile highlights the potential challenges of healthcare utilization (Table 1). A significant majority (85.09 %) of the women lacked formal education, and nearly all (98.69 %) were unemployed. Economic vulnerability was indicated by over 60 % of the population falling to the poorest to middle-wealth quintiles. High fertility patterns were prevalent, with 58.00 % having a birth order of four or higher and 58.10 % reporting a parity of four or more children. While over half of the population resided in urban areas, a substantial proportion was nomadic or rural. Limited media exposure (75.68 % ware Not Exposed) was observed.

**Table 1:** Socio-demographic and obstetric characteristics of pregnant women in the analytical sample (*n* = 4124).

Characteristic	Category	Frequency (Weighted N)	Percentage (%)
Age Group (Years)			
	15–19	280	6.79
	20–24	909	22.04
	25–29	1122	27.21
	30–34	819	19.86
	35–39	662	16.05
	40–44	252	6.11
	45–49	80	1.94
Educational Level			
	No Education	3509	85.09
	Primary	478	11.59
	Secondary	114	2.76
	Higher	23	0.56
Sex of Household Head			
	Male	2741	66.46
	Female	1383	33.54
Marital Status			
	Married	3709	89.94
	Divorced	263	6.38
	Widowed	152	3.69
Respondent Worked			
	Yes	54	1.31
	No	4070	98.69
Wealth Quintile			
	Poorest	844	20.47
	Poorer	1171	28.39
	Middle	888	21.53
	Richer	741	17.97
	Richest	480	11.64
Household Size			
	≤4 members	2208	53.54
	>4 members	1916	46.46
Birth Order			
	1	476	11.54
	2–3	1256	30.46
	≥4	2392	58.00
Place of Delivery			
	Health Facility	737	17.87
	Home	3387	82.13
Age of Household Head			
	<30	2565	62.20
	30–40	24	0.58
	41–54	145	3.52
	>54	1390	33.70
Parity			
	0–3 Children	1728	41.90
	4–7 Children	1807	43.82
	8+ Children	589	14.28
Region			
	Mudug	440	10.67
	Galgaduud	413	10.01
	Hiraan	392	9.51
	Middle Shabelle	416	10.09
	Banadir	858	20.80
	Bay	152	3.69
	Bakool	499	12.10
	Gedo	483	11.71
	Lower Juba	471	11.42
Residence			
	Rural	946	22.94
	Urban	2063	50.02
	Nomadic	1115	27.04
Media Exposure			
	Exposed	1003	24.32
	Not Exposed	3121	75.68

Media Exposure is defined as as “Exposed” if the respondent reported radio, television, or newspaper at least weekly; otherwise “Not Exposed. Wealth Quintiles are based on a PCA of household assets and classified into five equal population groups. Household Head is the person recognized as the head by other members.

## Bivariate analysis: factors associated with optimal ANC attendance

Table 2 presents the survey-adjusted distribution of optimal (≥4 visits) and suboptimal (<4 visits) ANC attendance across the predictor variable categories. Row percentages with 95 % confidence intervals and the design-based F-statistic (Rao-Scott adjusted Chi-square) assessing the association are shown for each variable. The survey-adjusted bivariate analysis indicated noteworthy associations between optimal ANC attendance and maternal educational level, marital status, household wealth quintile, place of last delivery, age of the household head, and administrative region.

**Table 2:** Bivariate associations between predictor variables and optimal ANC attendance (≥4 visits) (*n* = 4124).

		Number of ANC visits During Pregnancy		
Characteristic	Category	Suboptimal ANC % [95 % CI]	Optimal ANC % [95 % CI]	Design-based F (df), P-value
Age Group (Years)	15–19	95.6 [90.4, 98.0]	4.4 [2.0, 9.6]	F(4.94, 1270.76) = 0.83, *P* = 0.5
	20–24	94.2 [91.8, 95.9]	5.8 [4.1, 8.2]	
	25–29	93.2 [89.8, 95.5]	6.8 [4.5, 10.2]	
	30–34	94.0 [91.2, 95.9]	6.0 [4.1, 8.8]	
	35–39	95.6 [92.7, 97.4]	4.4 [2.6, 7.3]	
	40–44	96.1 [91.9, 98.1]	3.9 [1.9, 8.1]	
	45–49	93.2 [81.4, 97.7]	6.8 [2.3, 18.6]	
Educational Level	No Education	95.5 [93.8, 96.8]	4.5 [3.2, 6.2]	F(2.28, 586.65) = 13.74, *P* < 0.001
	Primary	90.1 [86.1, 93.1]	9.9 [6.9, 13.9]	
	Secondary	81.9 [66.2, 91.3]	18.1 [8.7, 33.8]	
	Higher	66.9 [35.1, 88.3]	33.1 [11.7, 64.9]	
Sex of Household Head	Male	94.1 [91.8, 95.7]	5.9 [4.3, 8.2]	F(1, 257) = 0.47, P = 0.5
	Female	94.8 [92.7, 96.3]	5.2 [3.7, 7.3]	
Marital Status	Married	94.7 [92.8, 96.1]	5.3 [3.9, 7.2]	F(1.89, 485.34) = 3.24, *P* = 0.04
	Divorced	90.1 [84.4, 93.8]	9.9 [6.2, 15.6]	
	Widowed	92.4 [83.4, 96.7]	7.6 [3.3, 16.6]	
Respondent Worked	Yes	92.6 [78.2, 97.7]	7.4 [2.3, 21.8]	F(1, 257) = 0.20, *P* = 0.7
	No	94.3 [92.4, 95.7]	5.7 [4.3, 7.6]	
Wealth Quintile	Poorest	99.8 [99.3, 99.9]	0.2 [0.1, 0.7]	F(3.04, 782.04) = 21.23, P < 0.001
	Poorer	97.9 [96.2, 98.8]	2.1 [1.2, 3.8]	
	Middle	91.9 [87.4, 94.8]	8.1 [5.2, 12.6]	
	Richer	91.6 [88.2, 94.1]	8.4 [5.9, 11.8]	
	Richest	87.8 [83.0, 91.4]	12.2 [8.6, 17.0]	
Household Size	≤4 members	93.9 [91.0, 95.9]	6.1 [4.1, 9.0]	F(1, 257) = 0.28, *P* = 0.6
	>4 members	94.7 [92.2, 96.4]	5.3 [3.6, 7.8]	
Birth Order	1	92.8 [87.8, 95.8]	7.2 [4.2, 12.2]	F(1.94, 498.83) = 2.74, *P* = 0.07
	2–3	95.7 [93.9, 97.1]	4.3 [2.9, 6.1]	
	≥4	93.8 [91.6, 95.4]	6.2 [4.6, 8.4]	
Place of Delivery	Health Facility	84.0 [78.8, 88.1]	16.0 [11.9, 21.2]	F(1, 257) = 52.13, *P* < 0.001
	Home	96.8 [95.3, 97.9]	3.2 [2.1, 4.7]	
Age of Household Head	<30	93.9 [91.6, 95.6]	6.1 [4.4, 8.4]	F(2.90, 744.08) = 2.96, *P* = 0.03
	30–40	84.7 [66.0, 94.0]	15.3 [6.0, 34.0]	
	41–54	94.2 [85.9, 97.7]	5.8 [2.3, 14.1]	
	>54	96.5 [95.0, 97.6]	3.5 [2.4, 5.0]	
Parity	0–3 Children	95.0 [92.8, 96.5]	5.0 [3.5, 7.2]	F(1.91, 489.84) = 1.92, *P* = 0.1
	4–7 Children	93.4 [90.8, 95.2]	6.6 [4.8, 9.2]	
	8+ Children	95.0 [92.6, 96.7]	5.0 [3.3, 7.4]	
Region	Mudug	97.3 [95.7, 98.4]	2.7 [1.6, 4.3]	F(4.69, 1206.01) = 3.36, *P* = 0.006
	Galgaduud	93.7 [88.5, 96.7]	6.3 [3.3, 11.5]	
	Hiraan	95.9 [92.9, 97.7]	4.1 [2.3, 7.1]	
	Middle Shabelle	93.7 [85.1, 97.5]	6.3 [2.5, 14.9]	
	Banadir	94.1 [90.2, 96.6]	5.9 [3.4, 9.8]	
	Bay	84.9 [71.4, 92.7]	15.1 [7.3, 28.6]	
	Bakool	96.5 [94.2, 97.9]	3.5 [2.1, 5.8]	
	Gedo	98.4 [96.0, 99.4]	1.6 [0.6, 4.0]	
	Lower Juba	96.0 [91.5, 98.1]	4.0 [1.9, 8.5]	
Residence	Rural	95.5 [91.9, 97.6]	4.5 [2.4, 8.1]	F(1.32, 339.56) = 1.85, *P* = 0.2
	Urban	93.5 [90.8, 95.4]	6.5 [4.6, 9.2]	
	Nomadic	97.0 [95.2, 98.1]	3.0 [1.9, 4.8]	
Media Exposure	Exposed	95.4 [92.6, 97.2]	4.6 [2.8, 7.4]	F(1, 257) = 2.77, P = 0.1
	Not Exposed	93.7 [92.0, 95.1]	6.3 [4.9, 8.0]	

CI = Confidence Interval. Design-based F is the Rao-Scott adjusted Chi-square test statistic. Bold P-values indicate statistical significance (p < 0.05).

Notably, strong positive gradients were observed for education and wealth, where the percentage of women achieving optimal ANC increased markedly with higher levels of education and wealth. Women who delivered in a health facility (16.0 % optimal ANC) were more likely to achieve optimal attendance than those who delivered at home (3.2 % optimal ANC). Significant regional variations were also confirmed, with the Bay region showing the highest percentage (15.1 %). Factors that were not significantly associated with optimal ANC attendance in the bivariate analysis included maternal age group, sex of the household head, respondents' work status, household size, birth order, parity, type of residence, and media exposure.

## Multivariable analysis: independent determinants of optimal anc attendance

A multivariable survey logistic regression model was employed to identify independent predictors of optimal ANC utilization (≥4 visits), adjusting for potential confounders. Table 3 presents both unadjusted odds ratios (cORs) and adjusted odds ratios (aORs), along with their 95 % confidence intervals (CIs) and p-values. Higher levels of maternal education were associated with increased odds of optimal ANC. Women with primary (cOR = 2.35, 95 % CI 1.48–3.72), secondary (cOR = 4.71, 95 % CI 1.97–11.29), and higher (cOR = 10.58, 95 % CI 2.80–39.97) education had significantly greater odds of optimal ANC than women with no education. A marked positive gradient was observed for household wealth. Compared to the poorest quintile, women in progressively wealthier quintiles exhibited drastically higher odds of optimal ANC, with cORs ranging from 8.75 (95 % CI 2.51–30.50) for the poorer quintile to 56.09 (95 % CI 17.41–180.69) for the richest. Home delivery was associated with significantly lower odds of optimal ANC (cOR = 0.17, 95 % CI 0.10–0.29) than facility deliveries. Several other factors showed significant but less pronounced associations. Women in Galgaduud (cOR = 2.46, 95 % CI 1.07–5.65), Banadir (cOR = 2.28, 95 % CI 1.07–4.85), and Bay (cOR = 6.53, 95 % CI 2.51–16.97) had higher optimal ANC odds versus women in Mudug.

**Table 3:** Multivariable logistic regression results for determinants of optimal ANC attendance (≥4 visits) (*n* = 4124).

Variable	Unadjusted			Adjusted		
	cOR	95 % CI	p-value	aOR	95 % CI	*p*-value
Age Group (Ref: 15–19)						
20–24	1.33	[0.55–3.20]	0.5	1.13	[0.41, 3.12]	0.8
25–29	1.59	[0.74–3.40]	0.2	1.48	[0.58, 3.79]	0.4
30–34	1.39	[0.58–3.32]	0.5	1.10	[0.37, 3.29]	0.9
35–39	0.99	[0.39–2.51]	0.9	0.90	[0.28, 2.88]	0.9
40–44	0.88	[0.29–2.71]	0.8	1.06	[0.30, 3.74]	0.9
45–49	1.57	[0.37–6.69]	0.5	1.90	[0.34, 10.43]	0.8
Education Level (Ref: No Education)						
Primary	2.35	[1.48–3.72]	<0.001	1.31	[0.84, 2.04]	0.2
Secondary	4.71	[1.97–11.29]	0.001	2.33	[1.01, 5.40]	0.05
Higher	10.58	[2.80–39.97]	0.001	5.36	[1.58, 18.15]	0.007
Sex of Household Head (Ref: Male)						
Female	0.88	[0.60–1.28]	0.5	0.90	[0.58, 1.39]	0.6
Marital Status (Ref: Married)						
Divorced	1.95	[1.12–3.38]	0.02	1.56	[0.82, 2.94]	0.2
Widowed	1.45	[0.61–3.45]	0.4	1.70	[0.69, 4.17]	0.2
Respondent Worked (Ref: No)						
Yes	0.75	[0.22–2.64]	0.7	0.72	[0.20–2.55]	0.6
Wealth Quintile (Ref: Poorest)						
Poorer	8.75	[2.51–30.50]	0.001	8.99	[2.54, 31.83]	0.001
Middle	35.90	[10.80–119.37]	<0.0001	24.19	[7.16, 81.77]	<0.0001
Richer	37.10	[11.62–118.49]	<0.0001	21.59	[6.44, 72.35]	<0.0001
Richest	59.35	[17.41–180.69]	<0.0001	29.66	[8.51, 101.27]	<0.0001
Family Size (Ref: ≤4)						
>4	0.86	[0.50–1.49]	0.6	0.90	[0.53–1.54]	0.7
Birth Order (Ref: 1)						
2–3	0.57	[0.34–0.97]	0.04	0.65	[0.37–1.16]	0.1
≥4	0.86	[0.51–1.44]	0.6	0.89	[0.39–1.95]	0.8
Place of Delivery (Ref: Health Facility)						
Home	0.17	[0.10–0.29]	<0.0001	0.29	[0.18–0.47]	<0.0001
Age of Household Head (Ref: <30)						
30–40	2.81	[0.97–8.14]	0.06	4.64	[1.65, 13.06]	0.004
41–54	0.96	[0.37–2.53]	0.9	1.03	[0.45, 2.34]	0.9
>54	0.56	[0.33–0.94]	0.03	0.77	[0.41, 1.48]	0.4
Parity (Ref: 0–3 Children)						
4–7 Children	1.35	[0.95–1.92]	0.09	1.53	[0.87, 2.70]	0.1
8+ Children	0.99	[0.60–1.63]	0.9	1.19	[0.60, 2.34]	0.6
Region (Ref: Mudug)						
Galgaduud	2.46	[1.07–5.65]	0.03	2.74	[1.13, 6.64]	0.03
Hiraan	1.55	[0.71–3.35]	0.3	0.88	[0.42, 1.84]	0.7
Middle Shabelle	2.47	[0.84–7.27]	0.1	1.34	[0.60, 2.98]	0.5
Banadir	2.28	[1.07–4.85]	0.03	1.27	[0.57, 2.83]	0.6
Bay	6.53	[2.51–16.97]	<0.001	4.07	[1.22, 13.60]	0.02
Bakool	1.34	[0.65–2.76]	0.4	0.70	[0.29, 1.67]	0.4
Gedo	0.59	[0.20–1.75]	0.3	0.76	[0.27, 2.10]	0.6
Lower Juba	1.54	[0.60–3.96]	0.4	1.00	[0.43, 2.35]	0.9
Residence (Ref: Rural)						
Urban	1.49	[0.71–3.10]	0.3	0.90	[0.45, 1.77]	0.8
Nomadic	0.66	[0.30–1.48]	0.3	1.42	[0.63, 3.20]	0.4

Ref: Reference Category (or Group), cOR: Crude Odds Ratio, aOR: Adjusted Odds Ratio, 95 % CI: 95 % Confidence Interval, p-value: Probability value, Bold text indicates a statistically significant.

Women with a birth order of 2–3 had lower odds of adequate ANC visits (cOR = 0.57, 95 % CI 0.34–0.97), while having a household head older than 54 years (cOR = 0.56, 95 % CI 0.33–0.94), women of higher parity (cOR = 1.35, 95 % CI 0.95–1.92), and no media exposure (cOR = 1.40, 95 % CI 0.94–2.08) were not significantly associated with optimal ANC. After adjusting for all variables, maternal education, wealth, place of delivery, region, and older household head age remained significant predictors of optimal ANC. Notably, the magnitude of the associations between maternal education and wealth was attenuated but remained robust. Secondary education (aOR = 2.33, 95 % CI 1.01–5.40) and higher education (aOR = 5.36, 95 % CI 1.58–18.15) still conferred greater odds of optimal ANC compared with no education.

The richest women maintained nearly 30 times the odds of optimal ANC relative to their poorest counterparts (aOR = 29.66, 95 % CI 8.51–101.27), although the adjusted odds ratios showed a gradient across wealth categories: Middle (aOR = 24.19, 95 % CI 7.16–81.77) and richer (aOR = 21.59, 95 % CI 6.44–72.35). The association between home delivery and lower odds of optimal ANC remained strong (aOR = 0.29, 95 % CI 0.18–0.47), as did the increased odds for women whose household heads were aged 30–40 years (aOR = 4.64, 95 % CI 1.65–13.06). Regional disparities persisted after adjustment, particularly in Bay (aOR = 4.07, 95 % CI 1.22–13.60) and Galgaduud (aOR = 2.74, 95 % CI 1.13–6.64). Variables such as maternal age, sex of the household head, marital status, respondent's work status, household size, birth order, parity, and type of residence were not significantly associated with optimal ANC attendance in the adjusted model.

## Discussion

This study aimed to identify the determinants of optimal ANC utilization, defined as four or more visits, among pregnant women in South and Central Somalia, using data from the 2020 Somalia Demographic and Ongoing conflict, instability, socioeconomic barriers, and limited healthcare infrastructure highlight the profound challenges faced by pregnant women in accessing maternal health services, likely influencing the low prevalence of optimal ANC visits in South and Central Somalia [15,16]. The findings revealed that the prevalence of optimal ANC attendance was exceptionally low at 5.7 % (95 % CI: 4.3 %–7.6 %), indicating that most pregnant women in these regions do not receive the previously recommended minimum number of ANC visits. This rate is considerably lower than those reported in neighboring countries such as Ethiopia (43 %) [17], and Tanzania (50.3 %) [18], as well as other LMICS such as Ghana (44.4 %) [19], Bangladesh (31.2 %) [20], and Nepal (70 %) [21].

Ongoing conflict, instability, socioeconomic barriers, and limited healthcare infrastructure highlight the profound challenges faced by pregnant women in accessing maternal health services, likely influencing the low prevalence of optimal ANC visits in South and Central Somalia. Maternal education emerged as a significant determinant, with women who had completed secondary education (aOR = 2.33, 95 % CI 1.01–5.40) or higher education (aOR = 5.36, 95 % CI 1.58–18.15) being considerably more likely to achieve optimal antenatal care (ANC) than those with no education. This positive association is well documented across low- and middle-income countries (LMICS) [17,18,20,22,23]. Possible explanations for this association include enhanced health literacy, a better understanding of preventive care benefits, increased female autonomy in healthcare decision-making, and an improved ability to navigate the healthcare system [24].

Furthermore, Household wealth has emerged as the most significant predictor of optimal ANC visits, demonstrating a clear and consistent relationship. Women from wealthier households were significantly more likely to attend optimal ANC than those from poorer backgrounds. This finding aligns with extensive research indicating that socioeconomic status is a fundamental determinant of healthcare access globally [18,[25], [26], [27], [28]]. Wealthier families are more likely to overcome financial barriers to healthcare, such as transportation and out-of-pocket costs, which often hinder access to timely, regular, and consistent services. Moreover, the place of delivery was significantly associated with ANC attendance. Women who delivered at home had lower odds (aOR = 0.29, 95 % CI 0.18–0.47) of receiving optimal ANC than those who delivered in health facilities. This finding is consistent with those of other studies [[29], [30], [31]]. ANC can facilitate facility delivery through counselling and risk identification, while common barriers, such as distance or cost, may hinder both [32].

Research has shown that mobile health clinics and community education campaigns can improve facility-based delivery [33,34]. Another important finding is that women with a head of household aged 30–40 exhibited notably higher odds (aOR = 4.64, 95 % CI 1.65–13.06) of achieving optimal ANC than those with heads younger than 30 years. Household leadership may reflect specific sociocultural or economic dynamics within Somalia, potentially indicating greater household stability or established resource management capabilities within the age group [18,20]. Furthermore, Significant regional disparities were identified, with women in the Galgaduud and Bay regions demonstrating a higher likelihood of attending optimal ANC than those in Mudug. These differences may be attributed to relatively stronger health infrastructure, greater humanitarian presence, and more stable security conditions in Bay and Galgaduud compared to Mudug, where conflict and service disruptions remain acute [35]. Such geographical variations are frequently observed in low- and middle-income countries (LMICS) [[36], [37], [38]].

Possible disparities include underlying inequalities in service availability, infrastructure quality, security, and local norms. Collectively, these gradients point to actionable responses: in addition to the demonstrated value of community education and mobile outreach, financial protection mechanisms (e.g., conditional cash transfers and transport vouchers) can offset direct and indirect costs for poorer households; long-term investment in girls' education can strengthen health literacy and autonomy in reproductive decisions; and improving the quality, cultural acceptability, and respectful nature of facility care can build trust and shift women who currently deliver at home toward skilled care throughout their pregnancy and childbirth. Further research should examine how health system readiness and the content/quality of ANC interact with women's perceptions of care across Somali regions, and longitudinal designs are needed to clarify causal pathways and evaluate the impact of policy interventions on ANC uptake over time. However, this study has several limitations. First, the cross-sectional design prevented the inference of causality, identifying only associations. Second, reliance on self-reported ANC attendance up to five years post-event introduced potential recall and social biases. Third, although the SDHS provides valuable population data, it does not capture crucial supply-side factors, such as the quality of care received or specific barriers faced at the facility level. Finally, focusing on South and Central Somalia limits the generalizability to other regions with distinct contexts.

## Conclusion

This analysis of the 2020 Somalia Demographic and Health Survey confirmed that optimal antenatal care remains extremely rare in South and Central Somalia, with fewer than six in every 100 women achieving at least four visits. Educational attainment and household wealth are the most powerful enablers of adequate care, suggesting that structural inequities rooted in poverty and limited schooling continue to constrain women's access to essential maternal health services. Delivering at home, an entrenched practice for most women, further signals restricted contact with skilled providers and compounds the risk of preventable complications. Regional differences and the age profile of household heads highlight that social context and decision-making power within families also shape care-seeking patterns. Closing these gaps will require a multi-pronged strategy that addresses both demand and supply.

## Ethics approval and consent to participate

This study utilized secondary data from the 2020 Somali Demographic and Health Survey (SDHS), conducted in accordance with established ethical guidelines. The research complied with ethical principles by obtaining the necessary approvals from the Somalia National Health Research Ethics Committee and the ICF Institutional Review Board. Informed consent was secured from all participants prior to data collection, ensuring that their rights and confidentiality were upheld throughout the research process.

## Consent for publication

Not applicable.

## Author's contributions

ASA Conceptualized this idea. The study design was developed collaboratively by ASA, MMA, MAO and YSAH. Material preparation and data collection were performed by YSAH and MMA. MAO analyzed and interpreted the data. The initial draft was composed by ASA, MMA and YSAH. All authors contributed to the writing, reviewing, and editing of the subsequent versions of the manuscript.

## Funding

The authors have not received any funding for this study.

## CRediT authorship contribution statement

**Mohamed Abdirahim Omar:** Writing – review & editing, Writing – original draft, Methodology, Formal analysis, Data curation. **Yahye Sheikh Abdulle Hassan:** Writing – review & editing, Writing – original draft. **Abdirasak Sharif Ali:** Writing – review & editing, Writing – original draft, Methodology. **Mohamed Mustaf Ahmed:** Writing – review & editing, Writing – original draft, Methodology.

## Declaration of competing interest

All authors declare that there is no conflict of interest with respect to this manuscript.
